# Outcomes of chemotherapy/chemoradiation vs. R2 surgical debulking vs. palliative care in nonresectable locally recurrent rectal cancer

**DOI:** 10.1177/03008916241253130

**Published:** 2024-05-10

**Authors:** Luca Sorrentino, Andrea Scardino, Luigi Battaglia, Raffaella Vigorito, Giovanna Sabella, Filippo Patti, Michele Prisciandaro, Elena Daveri, Alessandro Gronchi, Filiberto Belli, Marcello Guaglio

**Affiliations:** 1 Colorectal Surgery Unit, Fondazione IRCCS Istituto Nazionale dei Tumori, Milan, Italy; 2 Department of Radiology, Fondazione IRCCS Istituto Nazionale dei Tumori, Milan, Italy; 3 1st Pathology Division, Fondazione IRCCS Istituto Nazionale dei Tumori, Milan, Italy; 4 Radiation Oncology Unit, Fondazione IRCCS Istituto Nazionale dei Tumori, Milan, Italy; 5 Department of Medical Oncology, Fondazione IRCCS Istituto Nazionale dei Tumori, Milan, Italy; 6 Translational Immunology Unit, Fondazione IRCCS Istituto Nazionale dei Tumori, Milan, Italy; 7 Sarcoma Surgery Unit, Fondazione IRCCS Istituto Nazionale dei Tumori, Milan, Italy

**Keywords:** Locally recurrent rectal cancer, resectability, progression-free survival, chemoradiation, pelvic exenteration

## Abstract

Locally recurrent rectal cancer is resected with clear margins in only 50% of cases, and these patients achieve a three-year survival rate of 50%. Outcomes and therapeutic strategies for nonresectable locally recurrent rectal cancer have been much less explored. The aim of the study was to assess the three-year progression-free survival and the three-year overall survival in locally recurrent rectal cancer patients treated by chemotherapy/chemoradiation only vs. chemotherapy/chemoradiation and R2 surgical debulking vs. palliative care. A total of 86 patients affected by nonresectable locally recurrent rectal cancer were included: three-year progression-free survival was 15.8% with chemotherapy/chemoradiation vs. 20.3% with R2 surgical debulking (Log-rank p=0.567), but both rates were higher than best palliative care (0.0%, Log-rank p=0.0004). Three-year overall survival rates were respectively 62.0%, 70.8% and 0.0% (Log-rank p<0.0001). Chemotherapy/chemoradiation (HR 0.33, p=0.028) and R2 surgical debulking with or without chemotherapy/chemoradiation (HR 0.23, p=0.005) were independent predictors of improved progression-free survival on multivariate analysis. In conclusion, both chemotherapy/chemoradiation alone and R2 surgery with or without chemotherapy/chemoradiation provide a survival benefit over palliative care in nonresectable locally recurrent rectal cancer. However, considering that pelvic debulking is burdened by a high rate of complications, and considering its negligible impact on progression-free survival and overall survival when associated to medical therapy, surgery should be avoided in this setting.

## Introduction

Despite optimization of the multimodal cure of rectal cancer and careful surgery with total mesorectal excision, locally recurrent rectal cancer (LRRC) still occurs in about 6% of patients.^1,2^ LRRC represents a challenge for surgical oncologists, since it often requires wide exenterative surgery preceded by neoadjuvant pelvic re-irradiation with or without systemic chemotherapy.^3^ These heavy treatments are needed to minimize the likelihood of positive margins, which are the most impactful predictor of poor survival.^4^ However, LRRC are resected with clear margins in only 50% of cases.^5^ Several evidences are focused on patients treated with R0 surgery, and three-year survival is about 50%, while the outcomes and the appropriate treatments of nonresectable LRRC have been much less explored.^6^ The aim of the present study was to assess the three-year survival outcomes of nonresectable LRRC treated with different approaches.

## Methods

All consecutive patients affected by LRRC, treated at the Colorectal Surgery Unit of the National Cancer Institute of Milan (Italy) from 2008 to 2023, were retrospectively reviewed. Authorization from the Institutional Review Board was obtained prior to the study (SEBASTIAN project, protocol no. INT149/2019). Based on pelvic magnetic resonance imaging (MRI) at diagnosis, LRRC were classified according to the National Cancer Institute system: S1a for intraluminal relapses within the rectal stump or anastomotic wall; S1b for central, extraluminal relapses without involvement of regional organs; S1c for LRRC involving anterior genitourinary viscera; S2 in case of sacral involvement; S3 for lateral LRRC involving pelvic sidewall.^7^ Patients were all evaluated at the multidisciplinary Colorectal Tumor Board and underwent upfront surgery or neoadjuvant treatment based on pelvic localization, involvement of pelvic organs and previous treatments for primary rectal cancer (chemotherapy and/or pelvic irradiation). Neoadjuvant treatment consisted of pelvic (re)chemoradiation with a total dose of 30 to 54 Gy, depending on previous radiotherapy, delivered with 3D conformational hyperfractionated approach. Concurrent 5-fluorouracil (225 mg/m2 per day) or capecitabine (825 mg/m2 bid), seven days/week were administered. In case of advanced LRRC with extensive invasion of lateral pelvic sidewall, major vessels or bony pelvis, induction chemotherapy prior to neoadjuvant (re)chemoradiation was considered, mainly with 4-6 cycles of FOLFOX or FOLFIRI with a monoclonal antibody. After completion of neoadjuvant treatment, patients were re-staged with a pelvic MRI. Patients with resectable LRRC were recommended to exenterative surgery with curative intent. In case of persistently nonresectable LRRC evaluated on imaging, patients were directly addressed to continuation of systemic chemotherapy (if not contraindicated) without surgical resection, up to local and/or distant progression. In case of borderline resectability at re-staging, patients underwent explorative laparotomy and direct assessment of resectability with possible radical resection attempt or palliative surgery (colostomy, bowel bypass, etc.) depending on intraoperative findings. Patients with nonresectable LRRC and unfit for chemotherapy were addressed to best palliative care. Only patients with nonresectable LRRC were included for the analyses. The primary endpoints of the study were 1) the three-year progression-free survival (PFS) and 2) the three-year overall survival (OS) in LRRC patients treated by chemotherapy/chemoradiation only vs. chemotherapy/chemoradiation and R2 surgical debulking vs. palliative care. The impact of chosen therapeutic approach on three-year PFS and three-year OS was evaluated with the Kaplan-Meier survival method. Univariate and multivariate Cox regression analyses were performed to assess the independent role of therapeutic regimen on survival outcomes. Statistical significance was set at p<0.05 (two-tailed). Data analyses were performed using Prism version 9.0 (GraphPad Software Inc., California, USA).

## Results

**Figure 1.**
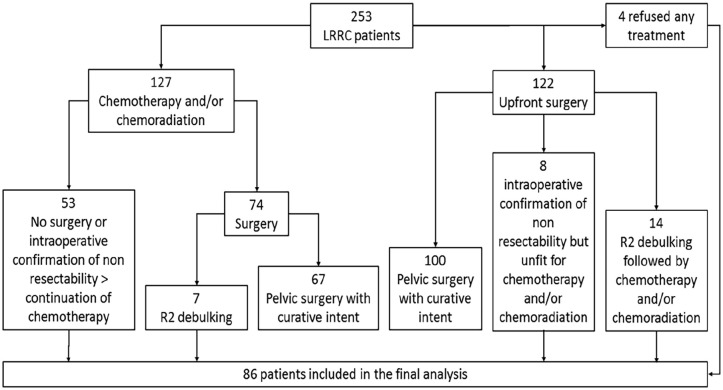
Study flow-chart.

As reported in Figure 1, among the 253 LRRC patients 127 were primarily treated with systemic chemotherapy with or without subsequent chemoradiation: in 53 cases surgery was not considered or nonresectability was confirmed at explorative laparotomy, thus these patients continued systemic chemotherapy. Of the 74 patients undergoing surgery after chemotherapy/chemoradiation, seven cases achieved R2 debulking only. Among 122 patients treated by upfront surgery, in eight cases nonresectability was confirmed and in other 14 cases R2 debulking was performed. Finally, four patients with nonresectable LRRC on imaging refused any active treatment, being addressed to follow up and best palliative care.

Thus, a total of 86 patients affected by nonresectable LRRC were included in the final analysis. Of these, 53 cases (61.6%) were treated by chemotherapy/chemoradiation without surgery, 21 (24.4%) were treated by R2 surgical debulking with or without chemotherapy/chemoradiation, and 12 (14.0%) were addressed to follow up and palliative care. Of the included patients, 26 (30.2%) were female and 60 (69.8%) male patients; mean age was 61.6 (±11.8) years. Mean time from treatment of primary rectal cancer and LRRC occurrence was 38.5 (±42.5) months. Mean size measured on baseline pelvic MRI was 45.0 (±26.4) mm. Pelvic localization of LRRC was central/anterior with involvement of genitourinary organs (S1b/S1c) in 24 (27.9%) patients, posterior/infiltrating the sacral bone (S2a/S2b) in 20 (23.3%), and lateral with involvement of pelvic sidewall in 42 cases (48.8%). Baseline characteristics of included patients are reported in Table 1. After surgical debulking, in eight cases (38.1%) a pelvic abscess occurred, leading to reoperation in one case (4.8%), and one patient experienced bowel occlusion treated conservatively. Mean post-operative hospitalization was 12.2 ±6.3 days.

**Table 1.** Baseline characteristics of included patients.

	Chemotherapy and/or chemoradiation (n=53)	Debulking ± chemotherapy and/or chemoradiation (n=21)	Palliative care (n=12)	P Value
**Age at diagnosis (years)**	59.2 (±10.6)	60.4 (±11.5)	73.9 (±10.5)	0.0002
**Gender**				0.874
Male	36 (67.9%)	15 (71.4%)	9 (75.0%)	
Female	17 (32.1%)	6 (28.6%)	3 (25.0%)	
**Time to LRRC (months)**	38.8 (±38.7)	46.7 (±58.0)	23.1 (±19.4)	0.312
**CEA (ng/mL)**	18.2 (±25.3)	36.6 (±122.0)	39.6 (±99.7)	0.591
**CA19.9 (U/mL)**	25.5 (±51.3)	27.9 (±40.0)	51.9 (±97.6)	0.469
**Size on MRI (mm)**	44.8 (±23.8)	40.0 (±26.7)	52.1 (±39.1)	0.667
**Localization of LRRC**				0.055
S1	10 (18.9%)	8 (38.1%)	6 (50.0%)	
S2	17 (32.1%)	2 (9.5%)	1 (8.3%)	
S3	26 (49.0%)	11 (52.4%)	5 (41.7%)	
**Chemotherapy regimen**				0.856
XELOX/FOLFOX ± anti-VEGF or anti-EGFR	34 (64.2%)	13 (61.9%)	-	
XELIRI/FOLFIRI ± anti-VEGF or anti-EGFR	19 (35.8%)	8 (38.1%)	-	
**KRAS Status**				0.655
Wild type	21 (70.0%)	11 (61.1%)	2 (50.0%)	
Mutated	9 (30.0%)	7 (38.9%)	2 (50.0%)	
Not available	23	3	8	

**Figure 2.**
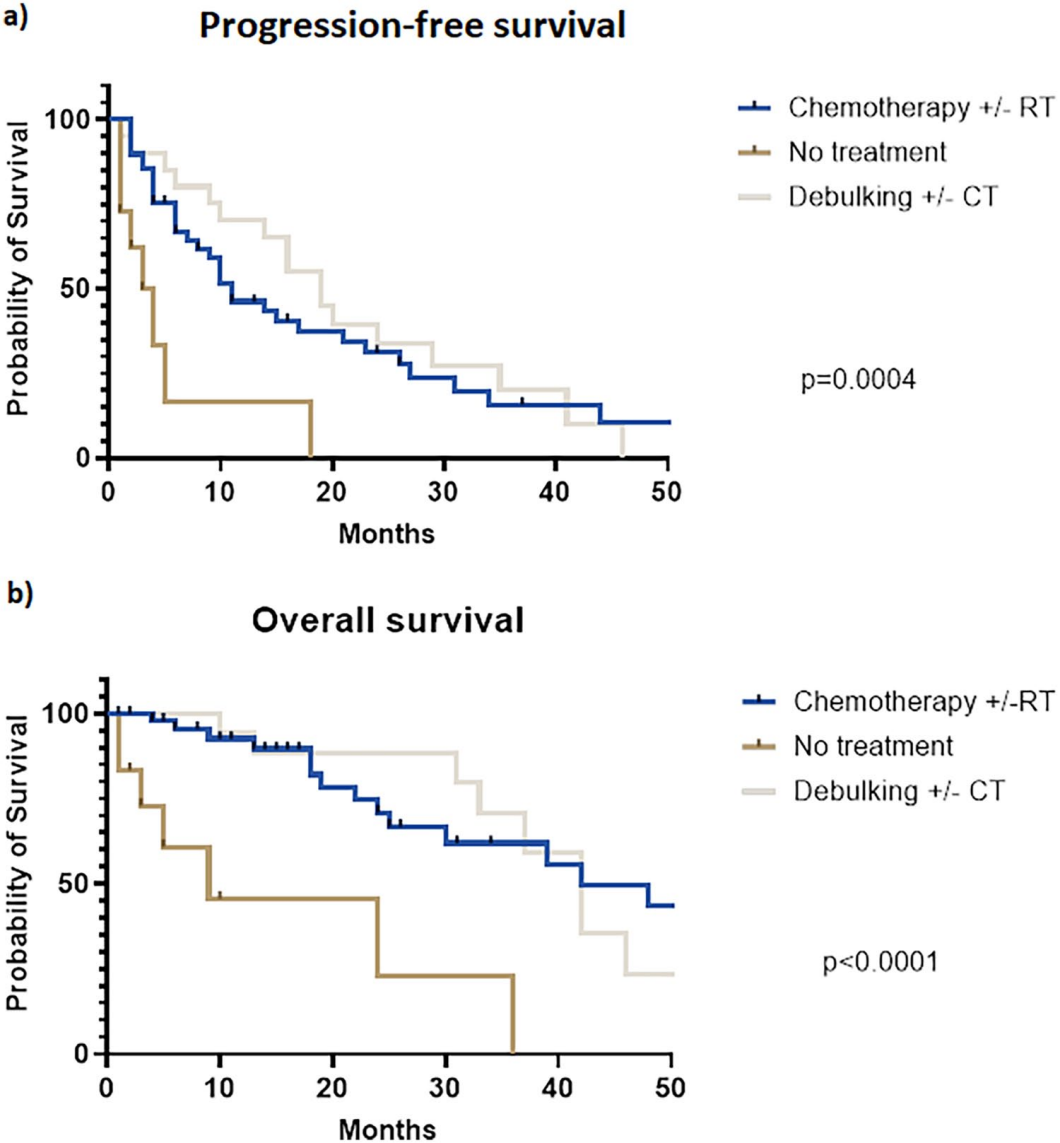
Progression-free survival (a) and overall survival (b) in patients affected by locally recurrent rectal cancer treated with chemotherapy/chemoradiation, surgical debulking with or without chemotherapy/chemoradiation, and palliative care.

Patients treated with chemotherapy/chemoradiation alone showed a three-year PFS similar to those undergoing chemotherapy/chemoradiation plus R2 surgical debulking (15.8% vs. 20.3%, Log-rank p=0.567), but both rates were significantly higher to best palliative care (3-yr PFS 0.0%, Log-rank p=0.0004), as reported in Figure 2a. Similarly, three-year OS rates were equal between chemotherapy/chemoradiation and R2 surgical debulking (62.0% vs. 70.8%, Log-rank p=0.829), but both were higher compared to no active treatment (three-year OS 0.0%, Log-rank p<0.0001, Figure 2b). On multivariate analysis, the use of chemotherapy/chemoradiation (HR 0.33, 95%CI 012-0.94, p=0.028) or even R2 surgical debulking (HR 0.23, 95%CI 0.08-0.68, p=0.005) were independent predictors of improved PFS compared to best palliative care (Table 2). The use of medical treatment or surgical debulking were also independent predictors of OS (respectively HR 0.19, p=0.003 and HR 0.15, p=0.004), as reported in Online Supplementary Table S1.

**Table 2.** Univariate and multivariate analyses for progression-free survival.

	Univariate analysis			Multivariate analysis		
	HR	95%CI	P value	HR	95%CI	P value
**Time to local recurrence**	1.00	0.99-1.01	0.976	-	-	-
**CEA**	1.00	0.99-1.01	0.85	-	-	-
**CA19.9**	1.01	1.00-1.01	0.009	1.00	1.00-1.01	0.126
**Size on MRI**	1.01	1.00-1.02	0.081	-	-	-
**Synchronous distant metastases**	1.32	0.68-2.36	0.383	1.48	0.61-3.33	0.358
**Pelvic localization of LRRC**						
S1a-b-c	(ref)	(ref)	(ref)	(ref)	(ref)	(ref)
S2a-b	0.82	0.40-1.64	0.58	1.51	0.57-3.90	0.393
S3	0.94	0.53-1.71	0.846	1.24	0.60-2.60	0.556
**Therapeutic approach**						
Chemotherapy ± radiotherapy	0.27	0.13-0.62	0.001	0.33	0.12-0.94	0.028
Debulking ± chemotherapy ± radiotherapy	0.21	0.09-0.53	0.001	0.23	0.08-0.68	0.005
No treatment	(ref)	(ref)	(ref)	(ref)	(ref)	(ref)
**KRAS status (mutated vs. wild-type)**	1.54	0.80-2.88	0.182	-	-	-
**Previous systemic chemotherapy**	1.66	0.94-3.00	0.085	-	-	-

## Discussion

The present study included only patients affected by nonresectable LRRC, thus focusing on a setting which normally is not considered by the majority of published studies. However, nonresectable lesions still represent about 50% of LRRC, and outcomes of these patients are particularly poor.^4,5^ Indeed, not only survival but also (and especially) quality of life are severely affected by nonresectable LRRC, with patients experiencing growing pelvic and/or sciatic pain refractory to analgesics.^8^ Therefore, exploring if different treatments affects the outcomes in this setting is particularly relevant.

According to the reported findings, both medical therapy with chemotherapy/chemoradiation or surgical treatment with non-radical debulking provided a benefit in PFS (Log-rank p=0.0004) and OS (Log-rank p<0.0001) compared to palliative care. While the positive effect of pelvic (re)irradiation or systemic chemotherapy on LRRC are well known,^9,10^ the observation that even R2 surgery provided a survival benefit was much more surprising. Indeed, non-radical surgery is notoriously the strongest predictor of worse survival outcomes in LRRC, leading to a five-year survival not exceeding 10%.^4^ A possible explanation is that surely radical surgery strongly improves survival compared to R2 resection, but at the same time R2 debulking is still better than no active treatment to prolong both PFS and OS.

No difference was found between the chemotherapy/chemoradiation and R2 surgical debulking in terms of PFS (respectively 15.8% vs. 20.3%) or OS (62.0% vs. 70.8%). Apparently, this finding could suggest that choosing non-radical debulking or chemotherapy/chemoradiation would provide the same benefit. However, it should be noted that 90.5% of patients undergoing R2 surgical debulking were also treated with neoadjuvant and/or adjuvant chemotherapy/chemoradiation. Thus, it could be assumed that surgical debulking added no survival benefit compared to medical treatment alone, and probably disease progression was mostly controlled by chemotherapy with or without chemoradiation rather than surgery.

Exenterative surgery and multivisceral resections for LRRC are associated with a high risk of post-operative complications and severe impairment of the quality of life.^11-13^ In the present study, up to 42.9% of patients treated with surgical debulking experienced a post-operative complication, mainly pelvic abscess (38.1%). Furthermore, recent evidences suggest that complex pelvic surgery carries a likelihood of major complications, defined as Clavien-Dindo grade III to V, up to 30%.^12^ In particular, pelvic abscess (12.8%) and sepsis (8.2%) were the most frequently reported major events. While the risk of these adverse events could be acceptable if R0 resection is achieved, considering the advantage in terms of survival, it should be cautiously considered prior to proceeding with surgical debulking in case of nonresectable LRRC. Also, medical treatment is not free from adverse effects: in a previous study, (re)chemoradiation was associated with pelvic abscess and sepsis or recto-vaginal/recto-vesical fistula in up to 15.2% of patients.^3^ However, since chemotherapy/chemoradiation positively affects local control and, rarely, could even lead to pathologic complete response of LRRC, its complications are much more acceptable for patients.

The present study has some limitations. First, it was a monocentric, retrospective study with a relatively small sample size. Secondly, study rationale is based on the concept of resectability, which is very heterogenous among institutions, thus findings of the present study could be not generalized for all centers. Third, patients treated with palliative care showed a significantly higher mean age (p=0.0002), which could have partially biased the survival analyses.

In conclusion, nonresectable LRRC represents an extremely challenging setting related to poor outcomes. Both chemotherapy/chemoradiation and R2 surgery provide a survival benefit over palliative care. However, considering that pelvic debulking is burdened by a high rate of complications and its impact on PFS and OS is probably negligible when associated to medical therapy, surgery should be avoided in this setting.

## Supplemental Material

sj-pdf-1-tmj-10.1177_03008916241253130 – Supplemental material for Outcomes of chemotherapy/chemoradiation vs. R2 surgical debulking vs. palliative care in nonresectable locally recurrent rectal cancer Supplemental material, sj-pdf-1-tmj-10.1177_03008916241253130 for Outcomes of chemotherapy/chemoradiation vs. R2 surgical debulking vs. palliative care in nonresectable locally recurrent rectal cancer by Luca Sorrentino, Andrea Scardino, Luigi Battaglia, Raffaella Vigorito, Giovanna Sabella, Filippo Patti, Michele Prisciandaro, Elena Daveri, Alessandro Gronchi, Filiberto Belli and Marcello Guaglio in Tumori Journal
