# Supplementary material for: Outcomes of chemotherapy/chemoradiation vs. R2 surgical debulking vs. palliative care in nonresectable locally recurrent rectal cancer
Source: Tumori. 2024 May 10;110(5):360–5. doi: 10.1177/03008916241253130 (PMC11459869; doi:10.1177/03008916241253130)
Supplement: sj-pdf-1-tmj-10.1177_03008916241253130 – Supplemental material for Outcomes of chemotherapy/chemoradiation vs. R2 surgical debulking vs. palliative care in nonresectable locally recurrent rectal cancer [file sj-pdf-1-tmj-10.1177_03008916241253130.pdf]

Outcomes of chemotherapy/chemoradiation vs. R2 surgical debulking vs. palliative care in non-resectable locally recurrent rectal cancer

Luca Sorrentino[a*#], MD, Andrea Scardino[a#], MD, Luigi Battaglia[a], MD, Raffaella Vigorito[b], MD, Giovanna Sabella[c], MD, Filippo Patti[d], MD, Michele Prisciandaro[e], MD, PhD, Elena Daveri[f], PhD, Alessandro Gronchi[g], MD, Filiberto Belli*[a], MD, Marcello Guaglio[a], MD

[a]Colorectal Surgery Unit, Fondazione IRCCS Istituto Nazionale dei Tumori, Milan, Italy

[b]Department of Radiology, Fondazione IRCCS Istituto Nazionale dei Tumori, Milan, Italy

[c]1st Pathology Division, Fondazione IRCCS Istituto Nazionale dei Tumori, Milan, Italy

[d]Radiation Oncology Unit, Fondazione IRCCS Istituto Nazionale dei Tumori, Milan, Italy

[e]Department of Medical Oncology, Fondazione IRCCS Istituto Nazionale dei Tumori, Milan, Italy

[f]Translational Immunology Unit, Fondazione IRCCS Istituto Nazionale dei Tumori, Milan, Italy

[g]Sarcoma Surgery Unit, Fondazione IRCCS Istituto Nazionale dei Tumori, Milan, Italy

Supplementary Tables

Supplementary Table S1. Univariate and multivariate analyses for overall survival

	Univariate analysis			Multivariate analysis		
	**HR**	**95%CI**	**P value**	**HR**	**95%CI**	**P value**
**Time to local recurrence**	1.00	0.98-1.01	0.446	-	-	-
**CEA**	1.00	0.99-1.01	0.291	-	-	-
**CA19.9**	1.01	1.00-1.01	0.14	1.01	0.99-1.01	0.245
**Size on MRI**	1.00	0.98-1.02	0.964	-	-	-
**Synchronous distant metastases**	1.21	0.45-2.77	0.676	1.49	0.43-4.48	0.495
**Pelvic localization of LRRC**						
S1a-b-c	(ref)	(ref)	(ref)	(ref)	(ref)	(ref)
S2a-b	0.52	0.19-1.29	0.168	0.64	0.19-2.03	0.447
S3	0.55	0.245-1.25	0.149	0.68	0.23-1.88	0.465
**Therapeutic approach**						
Chemotherapy ± radiotherapy	0.13	0.05-0.37	<0.001	0.19	0.06-0.61	0.003
Debulking ± chemotherapy ± radiotherapy	0.15	0.05-0.45	<0.001	0.15	0.04-0.55	0.004
No treatment	(ref)	(ref)	(ref)	(ref)	(ref)	(ref)
**KRAS status (mutated vs. wild-type)**	1.90	0.75-4.77	0.167	-	-	-
**Previous systemic chemotherapy**	2.36	1.01-6.16	0.059	-	-	-
